# Levels of hepatitis B antibody titers are affected by age and doses gap time in children from a high endemic area of the western Amazon

**DOI:** 10.1371/journal.pone.0253752

**Published:** 2021-07-01

**Authors:** Laura Cordeiro Gomes, Marina Cordeiro Gomes Sanson, Philip Brainin, Maria da Conceição Vieira de Melo, Rodrigo Medeiros de Souza, Janaína Mazaro, Karine Oliveira Lima, Júnia Silva Resende, Isabelle Victória Martins Vieira, Edinilson da Silva Mesquita, Luan Oliveira Matos, Isabelle Caroline Silva Dutra, Giuseppe Palmisano, Carsten Wrenger, Claudio Romero Farias Marinho, Rita do Socorro Uchôa da Silva

**Affiliations:** 1 Health and Sport Science Center, Federal University of Acre, Rio Branco, Acre, Brazil; 2 Multidisciplinary Center, Federal University of Acre, Cruzeiro do Sul, Acre, Brazil; 3 Herlev-Gentofte University Hospital, Hellerup, Denmark; 4 Laboratory of Public Health of Acre, Acre, Brazil; 5 Department of Parasitology, Institute of Biomedical Sciences, University of Sao Paulo, Sao Paulo, Brazil; Centre de Recherche en Cancerologie de Lyon, FRANCE

## Abstract

**Background:**

Despite completion of the vaccine schedule for hepatitis B virus (HBV), children may display levels of HBV surface antibodies (anti-HBs) that are considered inadequate for sufficient protection (<10 IU/L).

**Aims:**

Our aim was to investigate if age and gap time between HBV vaccine doses may negatively affect the levels of anti-HBs in children, and if these relationships are modified by sex.

**Methods:**

In a high-endemic HBV region of the western Brazilian Amazon we enrolled children who had completed the HBV vaccine schedule. All children underwent analysis of anti-HBs and a clinical examination.

**Results:**

We included 522 children (mean age 4.3 ± 0.8 years; 50% male). Median anti-HBs was 28.4 [interquartile range (IQR) 5.4 to 128.6] IU/L and 32% had anti-HBs <10 IU/L. The median gap time from last to preceding dose was 2.4 [IQR 2.1 to 3.3] months. Levels of anti-HBs decreased with higher age (-42% per year increase [95%CI -56% to -24%], p<0.001), but not with longer gap time (+23% per month increase [95%CI -16% to +62%], p = 0.249). After adjusting for relevant confounders, gap time became significant (p = 0.032) and age remained a significant predictor of anti-HBs (p<0.001).

**Conclusion:**

One third of assessed children displayed anti-HBs <10 IU/L. Levels of anti-HBs decreased with higher age and increased with longer gap time between the last two doses.

## Introduction

Infection with hepatitis B virus (HBV) is associated with significant morbidity and mortality, and constitutes a major public health challenge [1]. The virus may lead to both acute and/or chronic infection, potentially resulting in severe liver damage [1,2]. Approximately 50% of all people are considered to live in high-endemic areas where chronic HBV affects more than 8% of the population [3,4]. In Brazil, the occurrence of HBV is heterogeneously distributed [5], such that the western Brazilian Amazon has the highest endemicity in the country [6]. In addition, this region suffers from a high prevalence of hepatitis delta virus (HDV) [5,7,8], which is associated with super- and co-infections of patients already infected with HBV [8].

Brazil introduced the HBV vaccine program in 1989 for children <10 years old residing in high-endemic areas of the Amazon and in 1997 it was expanded to include all children below one year throughout the country [9]. The current Brazilian schedule for hepatitis B vaccine adopts four doses, of which the first is a monovalent hepatitis B vaccine given at birth and the others are pentavalent vaccines given at 2, 4 and 6 months. Each dose of the vaccine contains 10μg hepatitis B surface antigen (HBsAg) according to the World Health Organization (WHO) recommendation [10–13].

Transmission of HBV may occur parenterally, sexually and vertically (mother-child) [14–16] and about 90% of perinatally infected children become chronic carriers [4]. For children aged one to five years the risk of becoming a carrier decreases to 25–30%, while an immunocompetent adult has an approximate risk of 5% when encountering the virus [17]. Therefore, completion of the vaccine program is of great importance during childhood to ensure adequate protection. However, recent studies have suggested that the protective effect of the vaccine, as defined by the level of HBV surface antibodies (anti-HBs), may be affected by age and sex [18–20]. Several studies have demonstrated that anti-HBs may decrease with higher age [21–23] and how sex potentially modifies the immune response to viral vaccines [24,25]. Specifically, a greater humoral and cell-mediated immune response has been shown in female children and adults [25].

Although the state of Acre, located in the western Brazilian Amazon, has the highest prevalence and incidence of HBV in Brazil, and accounts for 25% of the reported cases of HDV [26], there is no current study being done to investigate HBV in children in this region of the Amazon. Hence, we aimed to investigate levels of anti-HBs in children who had completed the vaccination schedule and were from this region of the Amazon. We hypothesized that age and gap time between vaccine doses may affect levels of HBV antibodies and that these relationships are modified by sex [24,27].

## Material and methods

### Study subjects

This was a cross-sectional study conducted in Cruzeiro do Sul (Acre, Brazil), located in the western Amazon. We included children (age two to five years) born between 2014 and 2016 who commenced on the national vaccine schedule regardless of number of completed doses.

**Fig 1:**
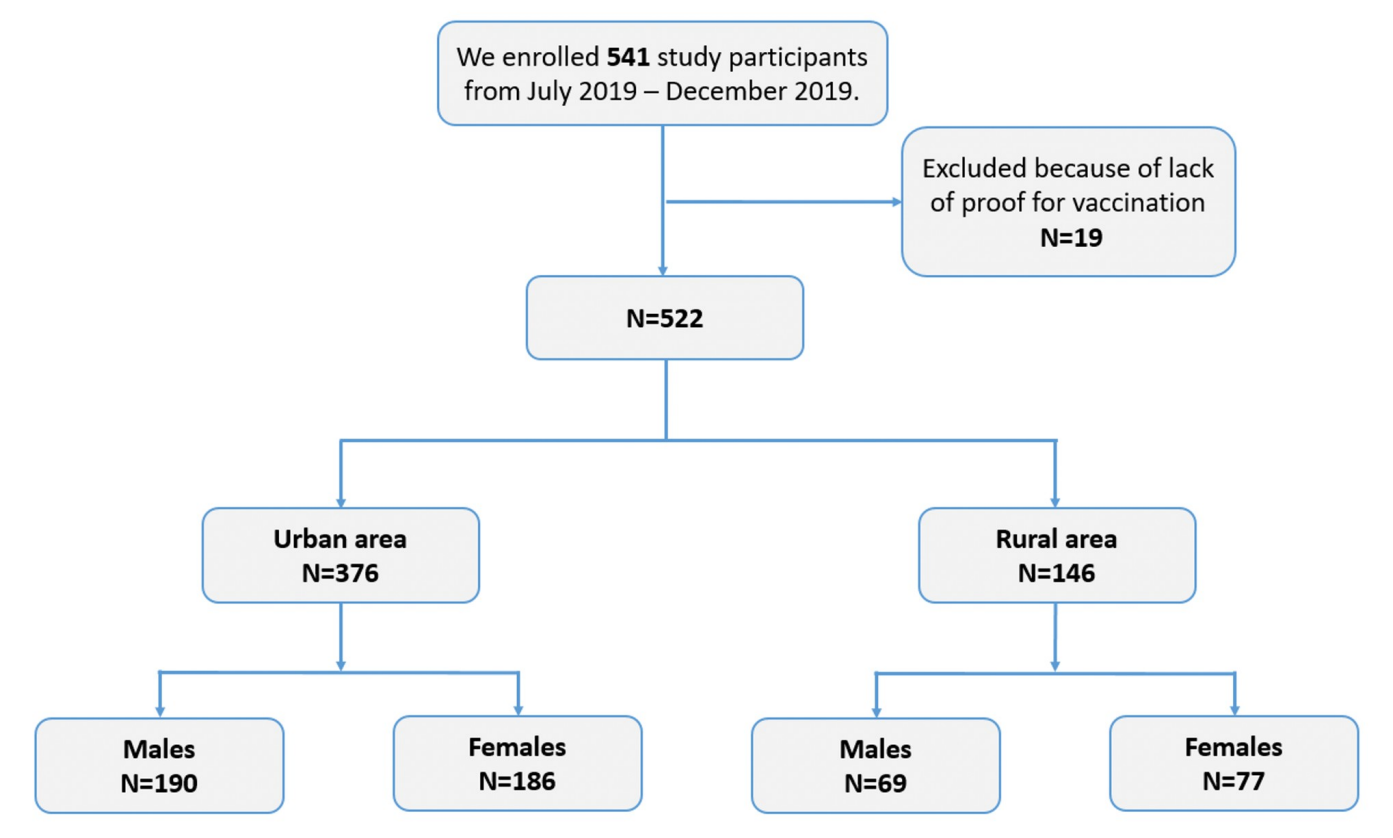
Study design flowchart. Consort diagram showing the inclusion of children in this study.

In collaboration with local healthcare units (n = 24) and kindergartens (n = 19), we selected study participants to be invited at random. Study sites were distributed between urban (n = 31) and rural (n = 12) areas. The distribution of participants enrolled in this study corresponded to the proportion of children associated with each healthcare unit/kindergarten in the city. Prior to study enrollment, parents and/or legal guardians were informed about the study, procedure and outcome. Because of limited access to healthcare units and lack of cellular network in rural areas, study participants in these areas (n = 146 children) were not randomly invited. Instead, healthcare professionals from each rural unit were responsible for inviting study participants. From July to December 2019, a total of 542 children were included in the study (Fig 1). Nineteen children were excluded due to lack of proof for vaccination, thus yielding a total study population of 522 participants.

### Study data

Children had height and weight measured and the body mass index (BMI) was calculated and classified according to age and sex specific guidelines defined by the WHO [28]. Information on vaccine status came from vaccine proofs and questionnaires.

### Analysis of HBV markers

All blood samples underwent centrifugation (2127 x *q*, 10 minutes) and were initially stored at -20°C at Federal University of Acre, Campus Floresta. Serological tests were performed at the Central Laboratory of Public Health of Acre. We conducted immunological analyses by commercial Chemiluminescent Microparticle Immunoassays (Architect System, Abbott Diagnostics, Sligo, Ireland or Wiesbaden, Germany) which were performed according to the manufacturer’s instructions. Three serological markers were analyzed: Anti-HBs [ARCHITECT Anti-HBs, limit of detection 0–1000 IU/L, specificity 99.67% [95%CI 99.22 to 99.89], sensitivity 97.54% [95%CI 95.97 to 98.62], HBsAg [ARCHITECT HBsAg Qualitative, specificity 100% [95%CI 99.41 to 100], sensitivity 99.80% [95%CI 98.90 to 99.99%] and antibody to hepatitis B core antigen (anti-HBc) [ARCHITECT Anti-HBc II, specificity 100% [95%CI 98.42 to 100], sensitivity 100% [95%CI 99.10 to 100%]. The first marker, anti-HBs, was assessed quantitatively and the latter markers qualitatively.

### Statistics

Continuous normally distributed variables were reported as mean values ± standard deviations and median [interquartile range (IQR)] was used for non-Gaussian distributed variables. Absolute and relative frequencies were described for categorical variables. Anti-HBs <10 IU/L, 10–99 IU/L and >100 IU/L and age <3, 3, 4 and ≥ 5 years old were used as defined categories. Characteristics of the participants stratified according to categories of anti-HBs were compared using Cuzick’s non-parametric test for trend and linear regression models. Pearson’s χ2 test was used for categorical variables, except when we had cells frequencies ≤ 5. In these cases, Fisher’s exact test was applied. Spearman’s test was used to examine the relationship between categories of anti-HBs and age.

We assessed the relationship between anti-HBs and age as well as anti-HBs and gap time between last and preceding vaccine dose. These relationships were assessed using linear regression models. We estimated the relative change in anti-HBs with 95% confidence interval (CI). In the same models we adjusted for (i) sex, (ii) mother’s HBsAg carrier status, (iii) number of total vaccine doses and (iv) living area (rural vs urban). A potential interaction with sex was examined in linear regression models. We considered two-sided p-values <0.050 as statistically significant. All statistical analyses were performed using Stata version 14.1 (StataCorp LP, College Station, TX).

### Ethics

The parents and/or legal guardians of all included study participants provided written informed consent according to the 2^nd^ Declaration of Helsinki. This study was approved by the Research Ethics Committee of the Acre State Hospital Foundation (CAAE: 09413619.8.0000.5009).

## Results

### Baseline data

**Fig 2:**
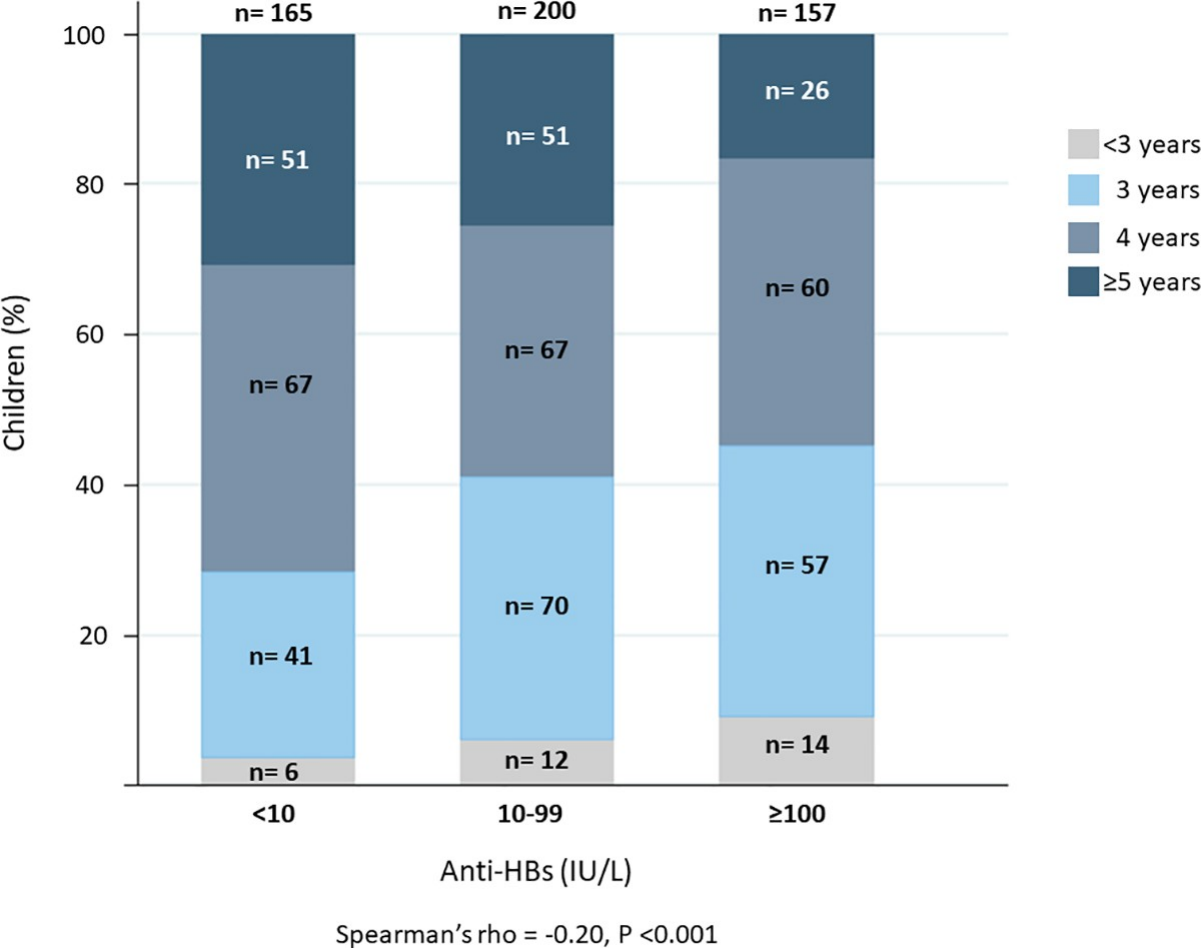
Anti-HBs titer categorized by age. Children were categorized by anti-HBs antibodies and age. Bar chart displaying number of children according to categories of anti-HBs (<10; 10–99; ≥100 IU/L) across the age (< 3; 3; 4; ≥5 years).

The study population included a total of 522 (50% males) children with a mean age of 4.3 ± 0.8 years (range 2.6 to 5.9 years), who were examined in 43 healthcare units and kindergartens in Cruzeiro do Sul, Acre, Brazil. The median level of anti-HBs was 28.4 [IQR 5.4 to 128.6] IU/L and 32% (n = 165) displayed levels of anti-HBs below 10 IU/L (Fig 2). All included children were negative to serology for HBsAg and anti-HBc. A total of 98% of the study population received ≥3 vaccines and median time from first to last vaccine dose was 7.3 [IQR 6.5 to 9.3] months.

When the population was stratified according to pre-defined categories of anti-HBs, age and time interval from last vaccination to examination date varied significantly across these categories (p trend<0.001; Table 1). No statistical difference was observed in sex, BMI, potential risk factors for HBV (sharing toothbrush, dental treatment, surgery, hospitalization) or parameters of socioeconomic status. When the population was stratified according to sex, only BMI differed significantly between males and females (S1 Table).

**Table 1:** Baseline information about children, their hepatitis B vaccine schedule and potential factor of risk to exposure to HBV, stratified by anti-HBs titer.

Categories of anti-HBs	Total	<10IU/L	10-99IU/L	≥100IU/L	*p* trend
N (%)	522 (100%)	165 (31.6%)	200 (38.3%)	157 (30.1%)	
**Baseline**					
Mean age, years	4.3 ±0.8	4.5 ±0.8	4.3 ± 0.9	4.1 ± 0.8	**<0.001**
Male, n(%)	259 (49.6%)	78 (47.3%)	99 (49.5%)	82 (52.2%)	0.670
BMI in percentiles*, n(%)					0.084
<25%	115 (22.0%)	25 (15.2%)	50 (25.3%)	40 (25.5%)	
25–75%	227 (43.5%)	81 (49.4%)	86 (43.4%)	60 (38.2%)	
>75%	177 (34.5%)	58 (35.4%)	62(31.3%)	57 (36.3%)	
Urban area, n(%)	376 (72.0%)	120 (72.7%)	142 (71.0%)	114 (72.6%)	0.920
Mother’s education*, n(%)					0.385
No education	9 (1.7%)	2 (1.2%)	6 (3.0%)	1 (0.6%)	
1–4 years	41 (7.9%)	12 (7.3%)	18 (9.0%)	11 (7.0%)	
5–8 years	102 (19.5%)	26 (15.8%)	41 (20.5%)	35 (22.3%)	
>8 years	369 (70.7%)	125 (75.8%)	135 (67.5%)	109 (69.4%)	
Father’s education*, years					0.200
No education	34 (6.5%)	7 (4.2%)	20 (10.0%)	7 (4.5%)	
1–4 years	54 (10.3%)	20 (12.1%)	16 (8.0%)	18 (11.5%)	
5–8 years	102 (19.5%)	31 (18.8%)	39 (19.5%)	32 (20.4%)	
>8 years	292 (55.9%)	97 (58.8%)	105 (52.5%)	90 (57.3%)	
Family income*, BRL					0.638
<1,000	243 (46.6%)	78 (47.3%)	92 (46.0%)	73 (46.5%)	
1,000–2,999	227 (43.5%)	73 (44.2%)	86 (43.0%)	68 (43.3%)	
3,000–4,999	20 (3.8%	5 (3.0%)	10 (5.0%)	5 (3.2%)	
>5,000	15 (2.9%)	7 (4.2%)	4 (2.0%)	4 (2.5%)	
**Vaccine**					
Number of vaccine doses					0.608
1	3 (0.6%)	1 (0.6%)	2 (1.0%)	0 (0.0%)	
2	9 (1.7%)	3 (1.8%)	5 (2.5%)	1 (0.6%)	
3	41 (7.9%)	16 (9.7%)	15 (7.5%)	10 (6.4%)	
4	469 (89.8%)	145 (87.9%)	178 (89.0%)	146 (93.0%)	
Gap time, months					
First to last dose	7.3 (6.5, 9.3)	7.1 (6.5, 8.6)	7.4 (6.5, 9.5)	7.4 (6.6, 9.6)	0.126
Last to preceding dose	2.4 (2.1, 3.3)	2.3 (2.0, 3.0)	2.5 (2.1, 3.6)	2.5 (2.1, 3.5)	0.114
Last dose to blood collection	43.7 (35.9, 51.7)	46.4 (38.3, 54.8)	44.0 (35.9, 51.9)	40.6 (32.9, 48.6)	**<0.001**
**Exposure**					
Degree of family with HBV*, n(%)					0.827
• 1^st^	23 (4.4%)	8 (4.8%)	9 (4.5%)	6 (3.8%)	
• 2^nd^	36 (6.9%)	13 (7.9%)	16 (8.0%)	7 (4.5%)	
• 3^rd^	45 (8.6%)	12 (7.3%)	17 (8.5%)	16 (10.2%)	
• 4^th^	12 (2.3%)	5 (3.0%)	4 (2.0%)	3 (1.9%)	
N/A	402 (77.0%)	127 (77.0%)	151 (75.5%)	124 (79.0%)	
HBsAg positive mother, n(%)					0.543
Yes	12 (2.3%)	4 (2.4%)	3 (1.5%)	5 (3.2%)	
No	510 (97.7%)	161 (97.6%)	197 (98.5%)	152 (96.8%)	
Toothbrush shared, n(%)					0.431
Yes	16 (3.1%)	7 (4.2%)	4 (2.0%)	5 (3.2%)	
No	506 (96.9%)	122 (73.9%)	166 (83.0%)	129 (82.2%)	
Dental treatment*, n(%)					0.150
Yes	102 (19.5%)	41 (24.8%)	33 (16.5%)	28 (17.8%)	
No	417 (79.9%)	122 (73.9%)	166 (83.0%)	129 (82.2%)	
Surgery*, n(%)					0.313
Yes	17 (3.3%)	8 (4.8%)	6 (3.0%)	3 (1.9%)	
No	504 (96.6%)	156 (94.5%)	194 (97.0%)	154 (98.1%)	
Hospitalization*, n(%)					0.590
Yes	150 (28.7%)	51 (30.9%)	55 (27.5%)	44 (28.0%)	
No	369 (70.7%)	114 (69.1%)	144 (72.0%)	111 (70.7%)	

BMI: Body mass index, BRL: Brazilian reais, HBV: Hepatitis B virus, N/A: Not applicable.

Non-normally distributed variables are presented as median [interquartile range].

* Variable with missing or not informed data.

### Antibody titers and age

**Fig 3:**
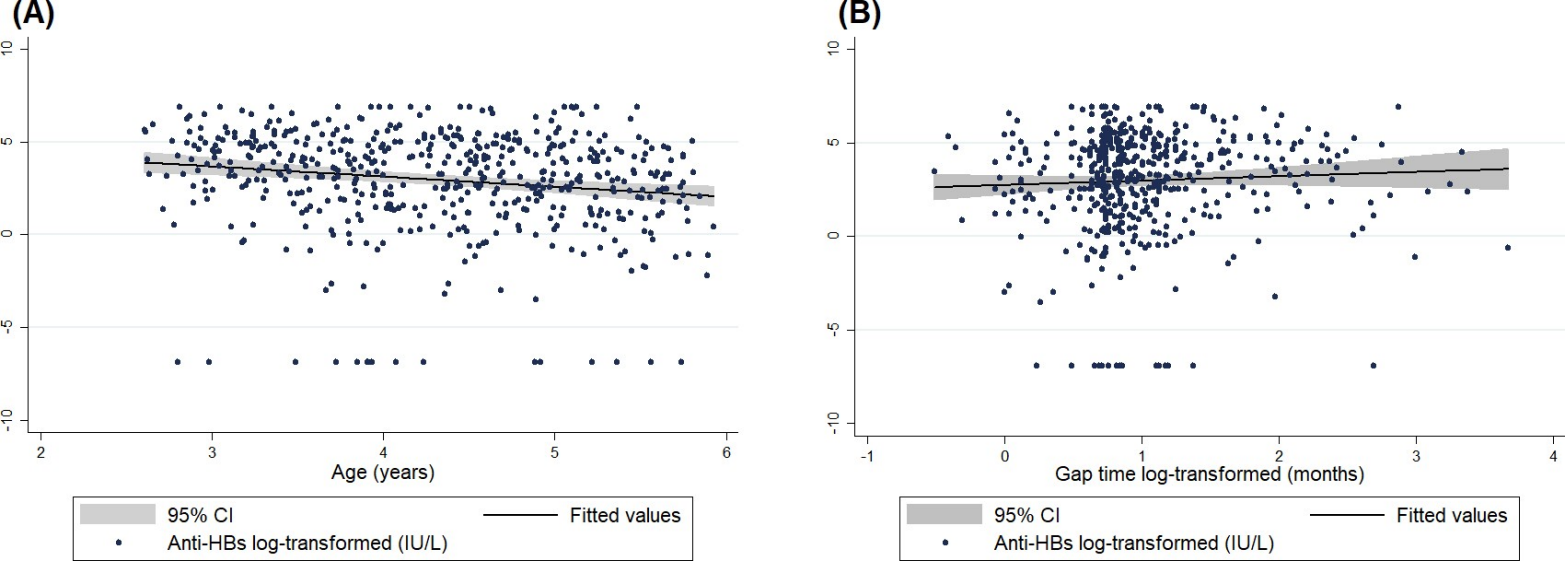
Association between Hepatitis B antibodies and (A) age, (B) gap time. (A) Linear regression model assessing anti-HBs (log-transformed) and age. Black line indicates association correlate, and gray lines indicate 95% CI. (B) Linear regression model assessing both log-transformed anti-HBs and gap time (months) between last and preceding vaccine dose. Black line indicates association correlate, and gray lines indicate 95% CI.

**Fig 4:**
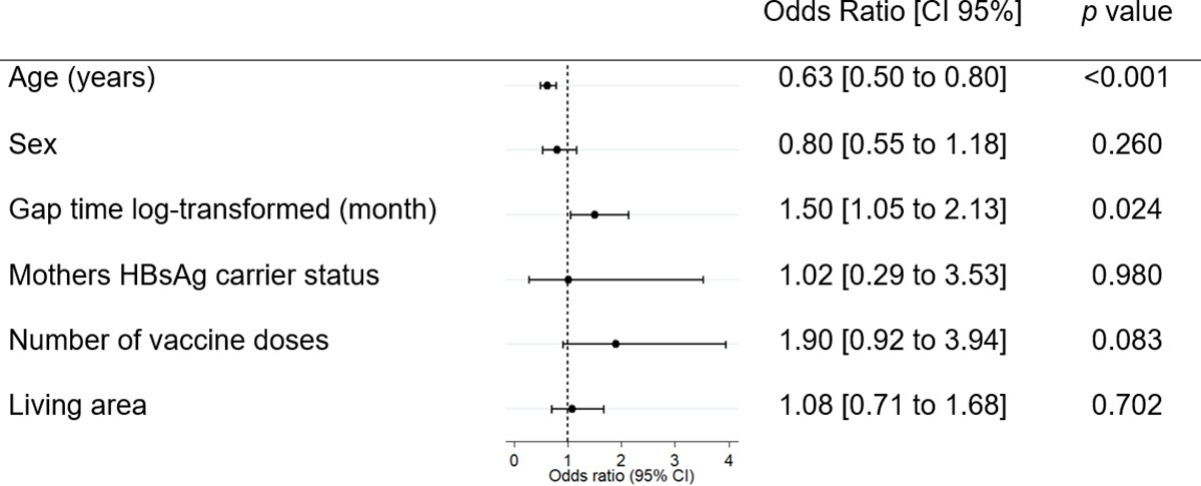
Risk of anti-HBs<10IU/L by factors associated with low anti-HB levels in a logistic regression model.

Children with age equal to or greater than five years old, 40% (n = 51) had anti-HBs <10 IU/L. Age categories differed significantly across categories of anti-HBs (Spearman’s rho = -0.20, p<0.001; Fig 2). When assessing anti-HBs as a continuous variable, anti-HBs decreased by 42% per year increase in age ([95%CI -56% to -24%], p<0.001; Fig 3A; Table 2). In multivariable analyses, the association with anti-HBs remained significant (43% decrease per year increase in age [95%CI -57% to -25%], p<0.001). This relationship between anti-HBs and age was not modified by sex (p = 0.425). In both, unadjusted and adjusted models anti-HBs data was log-transformed to have a normal distribution. The influence of age in antibodies was also found in a logistic regression model to assess risk of anti-HBs <10 UI/L (Odds ratio 0.63 [95%CI 0.50 to 0.80] p <0.001, Fig 4).

**Table 2:** Association between level of anti-HBs, age and gap time.

	Unadjusted		Adjusted	
	Coefficient (CI 95%)	*p* value	Coefficient (CI 95%)	*p* value
Age, per 1 year increase	- 42% (-56% to -24%)	<0.001	-43% (-57% to -25%)*	<0.001
Gap time(month), per 1%increase	+23% (-16% to +62%)	0.249	+45% (+4% to +87%)^†^	0.032

*Adjusted model included: Gap time between last and preceding vaccine dose, sex, mothers HBsAg carrier status, number of vaccine doses and living area.

^**†**^Adjusted model included: Age, sex, mothers HBsAg carrier status, number of vaccine doses and living area.

### Antibody titers and gap time

Median gap time between last and preceding vaccine dose was 2.4 [IQR 2.1 to 3.3] months. After log-transformation in anti-HBs and gap time, because their non-normal distribution, linear regression models were tested. No association between gap time and anti-HBs was found in unadjusted (+23%, 95% CI, -16% to +62%, p = 0.249), but it was detected in adjusted models (+45%, 95% CI, +4% to +87%, p = 0.032; Table 2; Fig 3B). Sex did not modify the relationship between anti-HBs and gap time of the last two doses (p = 0.949). The risk of anti-HBs <10 UI/L was influenced by this interval of doses (Odds ratio 1.50 [95%CI 1.05 to 2.13] p 0.024, Fig 4).

## Discussion

In the present study, we examined 522 children from Cruzeiro do Sul, Acre, Brazil, who had completed the HBV vaccine schedule. The key findings are that 32% of all children displayed anti-HBs <10IU/L and that the levels of anti-HBs decreased with higher age. Our findings are important because the study was conducted in a HBV endemic area of the Amazon basin, where adequate protection against HBV is of utmost importance [26].

An antibody concentration equal to or greater than 10 IU/L after completing the vaccination schedule is considered protective against HBV infection [29]. A recent meta-analysis by Mahmood et al. showed that two years after completion of the vaccination program in childhood, more than 95% of individuals had anti-HBs level ≥ 10 IU/L and this rate decreased to 70% eight years later [21]. By contrast, we examined children a mean time of 3.6 years after completion of the vaccine schedule and found that only two thirds of the study population achieved titers higher than 10 IU/L. Studies have already shown that children remain protected in childhood following completion of the vaccine schedule [20,22,23,30]. This is supported by our findings, where all children had negative HBsAg or anti-HBc profiles. However, it remains unclear whether the protection continues into adulthood despite decreasing antibodies [23,31].

While some studies have shown persistent protection up to 20 years after completion of the vaccine schedule [32–34], other studies have reported an increasing prevalence of HBV infection with higher age [35–38]. Specifically, one study found that in a population with high coverage of the vaccine schedule in childhood, the prevalence of HBV increased from 2.3% at age of 16 years to 8.4% at age 25 years old [37]. We found that levels of antibodies decreased with higher age, indicating that age potentially may influence the protective effect of the vaccine into adulthood.

As of today, the WHO does not support the need for a booster dose of HBV vaccine after completion of the primary vaccination schedule in immunization programmes [13]. Importantly, the response to a booster dose has been shown to decrease by age [18,23,39]. A rapid increase in anti-HBs after a challenge dose represents an anamnestic response, which is considered to indicate presence of immune memory generated after primary vaccination [39–41]. Meanwhile, it is still not elucidated whether an anamnestic response accurately reflects the immune memory [39]. The disappearance of an anamnestic effect with higher age has also been widely reported [19,33,42,43]. These findings explain why a booster dose may be of greater value if given during childhood or adolescence as compared to during adulthood.

Similarly, it has been demonstrated that low levels of anti-HBs may not ensure protection against all genotypes of the virus [44]. This is supported by *in vitro* studies, which have shown that low levels of vaccine-acquired anti-HBs were insufficient for neutralizing different genotypes of HBV [45,46]. In one study, polyclonal anti-HBs generated by immunization with the HBsAg genotype A vaccine inhibited only 60% of examined samples with HBsAg genotype C at titers of 30 IU/L [46]. In the present study, the median level of antibodies was 28.3 IU/L. These results are in line with another study by Stramer et al. which was carried out in blood donors in the United States [44]. The study detected six people who were HBV seronegative, but remained positive in the detection of virus DNA, despite they had been vaccinated with genotype A2 vaccine. Five of them had low or undetectable titers of anti-HBs. Only one person had been infected with the genotype from which the vaccine was derived (anti-HBs 3 IU/L) [44]. This suggests that anti-HBs in low concentrations may protect against infection by genotype A2, but that higher titers are required to ensure protection against other genotypes of the virus. In the high-endemic state of Acre, located in the Amazon basin, at least three genotypes of the hepatitis B virus have already been described [47].

The purpose of the HBV vaccine schedule is to provide adequate protection against HBV infection throughout child- and adulthood. Considering that we found overall low levels of anti-HBs, and that anti-HBs decreased significantly with age, in a high-endemic HBV and HDV area of the Amazon, this calls for novel attention on this topic.

However, children with a longer interval between the last two doses had higher levels of anti-HBs in this study. Due to this uncertainty of long-term protection after childhood vaccination, especially for a population that will have a higher risk of exposure during life, an extra dose would later be reasonable. In some European countries this is already done, the last dose is administrated being between 11 and 15 months of life, after a birth dose for high-risk infants and doses at 2, 3 and 4 months [23].

Although we had no long-term follow-up on HBV infection rate in the assessed population, further studies are encouraged to establish if the duration of protection generated by this vaccine extends into adulthood.

### Strengths and limitations

Study participants enrolled in rural areas were not randomized because of limited access to healthcare units and lack of cellular network. This could represent a potential source of bias in our study design. Although our multivariable models were adjusted for living area (urban vs rural), some children were vaccinated in both urban and rural areas. All questionnaires used to extract information from study participants involved self-reported information.

Some of the study participants received at least one vaccine dose delayed and twelve participants did not complete the schedule. As well, some study participants received 3 doses while others received 4 doses. This could potentially impact the levels of anti-HBs we measured. However, our multivariable models included this information.

Carrier status for HBsAg of the mothers were not tested in this study but was instead self-reported. A meta-analysis showed that the proportion of individuals with anti-HBs ≥10IU/L was more than twice as high for children with HBV positive mothers [14]. A close contact to the carrier mother during infancy is believed to work as a natural booster [14].

## Conclusion

In a high-endemic HBV area in the western Amazon, 32% of children who completed the HBV vaccine schedule had anti-HBs <10 IU/L. A vaccine schedule for HBV with an extra dose in later years of childhood, might be considerate as a strategy to ensure protective anti-HBs levels in endemic area. Future studies should address whether levels of anti-HBs <10IU/L, measured during childhood, provide adequate protection against HBV into adulthood.

## Supporting information

S1 Table Baseline information stratified by sex. BMI: Body mass index, BRL: Brazilian reais, HBV: Hepatitis B virus, N/A: Not applicable. Non-normally distributed variables are presented as median [interquartile range]. * Variable with missing or not informed data.
(PDF)

S1 File (XLS)
