# Supplementary material for: Levels of hepatitis B antibody titers are affected by age and doses gap time in children from a high endemic area of the western Amazon
Source: PLoS One. 2021 Jul 1;16(7):e0253752. doi: 10.1371/journal.pone.0253752 (PMC8248698; doi:10.1371/journal.pone.0253752)
Supplement: S1 Table — BMI: Body mass index, BRL: Brazilian reais, HBV: Hepatitis B virus, N/A: Not applicable. Non-normally distributed variables are presented as median [interquartile range]. * Variable with missing or not informed data. (PDF) [file pone.0253752.s001.pdf]

S1 Table. Baseline information stratified by sex.

	Male	Female	P trend
N (%)	259	263	
**Baseline**			
Anti-HBs, IU/L	28.7 (6.3, 133.9)	27.5 (5.2, 124.3)	0.502
Mean age, years	4.4 ±0.8	4.3 ±0.8	0.203
BMI in percentiles*, n(%)			**0.036**
<25%	58 (22.6%)	57 (21.8%)	
25-75%	99 (38.5%)	128 (48.9%)	
>75%	100 (38.9%)	77 (29.4%)	
Urban area, n(%)	190 (73.4%)	186 (70.7%)	0.500
Mother's education*, n(%)			0.958
No education	4 (1.5%)	5 (1.9%)	
1-4 years	19 (7.3%)	22 (8.4%)	
5-8 years	51 (19.7%)	51 (19.4%)	
>8 years	184 (71.0%)	185 (70.3%)	
Father's education*, years			0.570
No education	14 (5.4%)	20 (7.6%)	
1-4 years	28 (10.8%)	26 (9.9%)	
5-8 years	45 (17.4%)	57 (21.7%)	
>8 years	151 (58.3%)	141 (53.6%)	
Family income*, BRL			0.880
<1,000	117 (45.2%)	126 (47.9%)	
1,000 – 2,999	117 (45.2%)	110 (41.8%)	
3,000 – 4,999	10 (3.9%)	10 (3.8%)	
>5,000	6 (2.3%)	9 (3.4%)	
**Vaccine**			
Number of vaccine doses			0.851
1	2 (0.8%)	1 (0.4%)	
2	4 (1.5%)	5 (1.9%)	
3	22 (8.5%)	19 (7.2%)	
4	231 (89.2%)	238 (90.5%)	
Gap time, months			
First to last dose	7.2 (6.5, 9.1)	7.4 (6.5, 9.3)	0.356
Last to preceding dose	2.3 (2.0, 3.1)	2.4 (2.1, 3.5)	0.151
Last dose to blood collection	44.3 (36.6, 51.9)	43.3 (35.4, 51.5)	0.209
**Exposure**			
Degree of family with HBV*, n(%)			0.382
- 1st	10 (3.9%)	13 (4.9%)	
- 2nd	21 (8.1%)	15 (5.7%)	
- 3rd	17 (6.6%)	28 (10.6%)	
- 4th	7 (2.7%)	5 (1.9%)	
N/A	201 (77.6%)	201 (76.4%)	
HBsAg positive mother, n(%)			0.382
Yes	4 (1.5%)	8 (3.0%)	
No	255 (98.5%)	255 (97.0%)	
Toothbrush shared, n(%)			0.980
Yes	8 (3.1%)	8 (3.0%)	
No	251 (96.9%)	255 (97.0%)	
Dental treatment*, n(%)			0.220
Yes	50 (19.3%)	52 (19.8%)	
No	209 (80.7%)	208 (79.1%)	
Surgery*, n(%)			0.470
Yes	7 (2.7%)	10 (3.8%)	

No	251 (96.9%)	253 (96.2%)	
Hospitalization*, n(%)			0.390
Yes	81 (31.3%)	69 (26.2%)	
No	177 (68.3%)	192 (73.0%)	

BMI: body mass index, BRL: Brazilian reais, HBV: hepatitis B virus, N/A: not applicable.
Non-normally distributed variables are presented as median [interquartile range].

* Variable with missing or not informed data.
